# Reducing household air pollution exposure to improve early child growth and development; a randomized control trial protocol for the “Poriborton-Extension: The CHANge trial”

**DOI:** 10.1186/s13063-022-06342-5

**Published:** 2022-06-16

**Authors:** Camille Raynes-Greenow, Sk Masum Billah, Sajia Islam, S. M. Rokonuzzaman, Fahmida Tofail, Elizabeth K. Kirkwood, Ashraful Alam, Ryan Chartier, Tarana E. Ferdous, Shams El Arifeen, Michael J. Dibley, Nusrat Homaira, Alison Hayes, Jonathan Thornburg, Patrick Kelly

**Affiliations:** 1 The University of Sydney, School of Public Health, Sydney, Australia; 2 Maternal and Child Health Division, icddr,b, Dhaka, Bangladesh; 3 Nutrition and Clinical Services Division, icddr,b, Dhaka, Bangladesh; 4 RTI International, Research Triangle Park, NC 27707 USA; 5 UNSW, Sydney, Kensington, Australia

**Keywords:** Household air pollution, Perinatal mortality, Bangladesh, Cluster randomized controlled trial, Child development

## Abstract

**Background:**

Globally, household air pollution (HAP) is a leading environmental cause of morbidity and mortality. Our trial aims to assess the impact of liquefied petroleum gas (LPG) for cooking to reduce household air pollution exposure on child health outcomes, compared to usual cooking practices in Bangladesh. The primary aim is to evaluate if reduced exposure to HAP through the provision of LPG for cooking from early gestation through to age 2 improves child anthropometry, health, and neuro-cognitive developmental outcomes, compared to children exposed to emissions from usual practice.

**Methods:**

Two-arm parallel cluster randomized controlled trial (cCRT). We will extend the intervention and follow-up of our existing “Poriborton” trial. In a subset of the original surviving participants, we will supply LPG cylinders and LPG stoves (intervention) compared to usual cooking practices and extend the follow-up to 24 months of age. The expected final sample size, for both (intervention and control) is 1854 children with follow-up to 2 years of age available for analysis.

**Discussion:**

This trial will answer important research gaps related to HAP and child health and neuro-cognitive developmental outcomes. This evidence will help to understand the impact of a HAP intervention on child health to inform policies for the adoption of clean fuel in Bangladesh and other similar settings.

**Trial registration:**

The Poriborton: Change trial: Household Air Pollution and Perinatal and early Neonatal mortality is registered with the Australian New Zealand Clinical Trials Registry, ACTRN12618001214224, original trial registered on 19th July 2018, extension approved on 23rd June 2021. www.anzctr.org.au.

## Administrative information

Note: the numbers in curly brackets in this protocol refer to SPIRIT checklist item numbers [1]. The order of the items has been modified to group similar items (see http://www.equator-network.org/reporting-guidelines/spirit-2013-statement-defining-standard-protocol-items-for-clinical-trials/).

**Table:** 

Title {1}	Reducing household air pollution exposure to improve early child growth and development; a randomized control trial protocol for the “Poriborton-Extension: The CHANge trial”.
Trial registration {2a and 2b}.	The Poriborton: Change trial: Household Air Pollution and Perinatal and early Neonatal mortality is registered with the Australian New Zealand Clinical Trials Registry, ACTRN12618001214224, original trial registered on 19th July 2018, extension approved on 23rd June 2021. www.anzctr.org.au.
Protocol version {3}	PR-17103/Version 3 / 9th July 2021
Funding {4}	The Poriborton-Extension Trial is funded by the National Health and Medical Research Council of Australia (GNT_2001264). The trial sponsor is The University of Sydney, and contact details are available from the corresponding author. The funders do not have any role in the study design, data collection and interpretation of data.
Author details {5a}	*Camille Raynes-Greenow*^1^* The University of Sydney, School of Public Health. Australia (*corresponding author) *Sk Masum Billah*^*1&2*^*The University of Sydney, School of Public Health. Australia and Maternal and Child Health Division, icddr,b Bangladesh* *Sajia Islam*^*2*^*Maternal and Child Health Division, icddr,b Bangladesh* *SM Rokonuzzaman*^*2*^*Maternal and Child Health Division, icddr,b Bangladesh* *Fahmida Tofail*^*2*^*Nutrition and Clinical Services Division, icddr,b Bangladesh* *Elizabeth K Kirkwood*^*1*^*The University of Sydney, School of Public Health. Australia* *Ashraful Alam*^*1*^*The University of Sydney, School of Public Health. Australia* *Ryan Chartier*^*4*^*RTI International, Research Triangle Park, NC 27707, USA* *Tarana E Ferdous*^*2*^*Maternal and Child Health Division, icddr,b Bangladesh* *Shams El Arifeen*^*2*^*Maternal and Child Health Division, icddr,b Bangladesh* *Michael J Dibley*^*1*^*The University of Sydney, School of Public Health, Australia.* *Nusrat Homaira*^*3*^*University of New South Wales, Australia.* *Alison Hayes*^*1*^*The University of Sydney, School of Public Health, Australia* *Jonathan Thornburg*^*4*^*RTI International, Research Triangle Park, NC 27707, USA* *Patrick Kelly*^*1*^*The University of Sydney, School of Public Health. Australia*
Name and contact information for the trial sponsor {5b}	Camille Raynes-Greenow camille.raynes-greenow@sydney.edu.au The University of Sydney, School of Public Health. Australia.
Role of sponsor {5c}	The trial sponsor is The University of Sydney and they do not have any role in the study design, data collection and interpretation of data

## Introduction

### Background and rationale {6a}

Globally, household air pollution (HAP) is one of the leading environmental causes of death and adverse health outcomes [2]. HAP consists of emissions from burning solid fuels for cooking or heating that contain toxic respirable particles, including particulate matter (PM2.5, < 2.5 μm in diameter, and PM10 < 10 μm) [3]. Approximately 3.8 billion people or 49% of the world’s population, mostly poor people from low- and middle-income countries (LMIC), are still exposed to HAP from the use of solid fuels for cooking and heating, contributing to over 4 million premature deaths [2, 4]. Measurement of PM2.5 in households using solid fuels is at least 10 to 50 times in excess of the safe levels set by the WHO. Women and children are particularly at risk due to a combination of their higher vulnerability and chronic levels of exposure due to the gendered division of labor and domestic responsibilities [5]. The use of inefficient stoves in poorly ventilated areas and long hours cooking and heating cause exposure to HAP [3]. Children under the age of five are more likely to be exposed to HAP, staying close to their mothers near the domestic hearth.

Over 149 million children under five suffered from stunting in 2020 [6], and HAP is considered to account for a large proportion of stunting [7, 8]. Stunting is defined as height-for-age 2 standard deviations below the growth standards, and stunted children have both physical and cognitive developmental delays. Few studies examine the biological pathways by which HAP causes stunting [9]. It may be through the same pathway that exposure to environmental tobacco smoke reduces birth weight, thus contributing to stunting [10, 11], or through acute respiratory infections [12], or anemia [13], all of which are associated with stunting. During the antenatal period, exposure to air pollution may induce oxidative stress or systematic inflammation that occurs, as a result, may be a pathway to growth retardation [9]. Exposure to air pollution during the postnatal period may promote stunting via impairments in the immune system and development, clinical and subclinical infections, changes in nutrition (dietary intake and metabolism), and altered bone metabolism [9]. The epidemiological evidence is from retrospective observational studies. A recent review of solid fuel use on stunting ascertained that those children living in homes that used solid fuels as the primary fuel source were three times more likely to be severely stunted compared to those in households using cleaner fuel, such as liquefied petroleum gas (LPG) [12]. In a population-based study in India, children exposed to solid fuel compared to those unexposed had differences in mean height-for-age *z*-scores of 0.61, a 21.3% difference after accounting for confounding [14]. Further research from India revealed households using clean cooking fuels had reduced the likelihood of stunting after controlling for socioeconomic and demographic factors [8]. However, there have been no randomized controlled trials of HAP interventions and stunting, which this study aims to address.

HAP is a recognized lung health hazard, and early life exposure determines the lung function trajectory [15, 16]. The underlying cause of many acute lower respiratory infections (ALRI) deaths in children may be due to immature respiratory and immune systems and exposure to inefficient burning of solid fuels in households [5, 17–19]. Infants exposed to HAP experience ALRI’s in early life and are associated with reduced lung function and the persistence of respiratory symptoms [20]. Children living in households using solid fuel for cooking are far more as likely to develop ALRI compared to children in households using clean fuels [17, 21]. This suggests that early intervention is critical to improve lung health.

Evidence of the effect on child development and specifically on neuro-developmental delays from ambient air pollution literature has been accumulating [10–12, 22]. In a review of air pollution and cognitive outcomes, four included studies examined in utero exposure and follow-up ≤ 5 years of age, and eight studies examined postnatal exposure [14]. Pollution resulted in delays in motor, language and social skills at ages 6 and 18 months, and the postnatal exposure studies found effects mostly in boys. The studies were heterogeneous (design, population, source/type of exposure, follow-up), and all were observational. PM2.5 was specifically implicated in children aged 15 months; with every 1 μg/m3 increase in PM2.5, there were significant decreases in the mental score of the Bayley Scales of Infant Development (*β* = − 0.39 with PM2.5 exposure) [23].

To our knowledge, Poriborton is the largest trial specifically investigating reduced exposure to HAP and perinatal outcomes. This extension of Poriborton will make it one of the longest intervention and follow-up periods of the trials in this field. It specifically addresses identified research priorities related to HAP, including investigating the risk of severe infection, child growth, and cognitive development [8]. This trial will help to understand the impact of a HAP intervention on child health using existing research infrastructure and produce high-quality evidence.

### Objectives {7}

The primary aim is to evaluate if reduced exposure to HAP through the provision of LPG for cooking from early gestation through to age 2 improves child growth, respiratory illness, and neuro-cognitive developmental outcomes. Specifically, we aim to evaluate if reduced exposure to HAP to age 2 reduces stunting by 20% (28% in intervention and 35% control), significantly reduces the prevalence and severity of acute lower respiratory infections, improves child development by 0.3 SD at 24 months of age, and is a cost-effective intervention to deliver to the community considering the return to investment.

### Trial design {8}

The study design is a two-arm cluster randomized controlled superiority trial (cRCT). We will use the existing trial infrastructure of Poriborton trial, which provided LPG cylinders and LPG stoves from early pregnancy until birth, and the control arm continued usual cooking practices. The usual cooking practice in this setting is a traditional clay stove with various biomass fuels. For further details, see the Poriborton trial protocol [24]. We will use the existing trial infrastructure of Poriborton and seamlessly continue the LPG cylinder provision (intervention), extending the follow-up to 24 months of child’s age in the intervention arm. The intervention of the extension will commence around the time of birth (once the most recent cylinder needs replacing but not before seven completed days post-partum), and hence, we will have uninterrupted LPG coverage up to 2 years of age.

## Methods: participants, interventions, and outcomes

### Study setting {9}

**Fig. 1:**
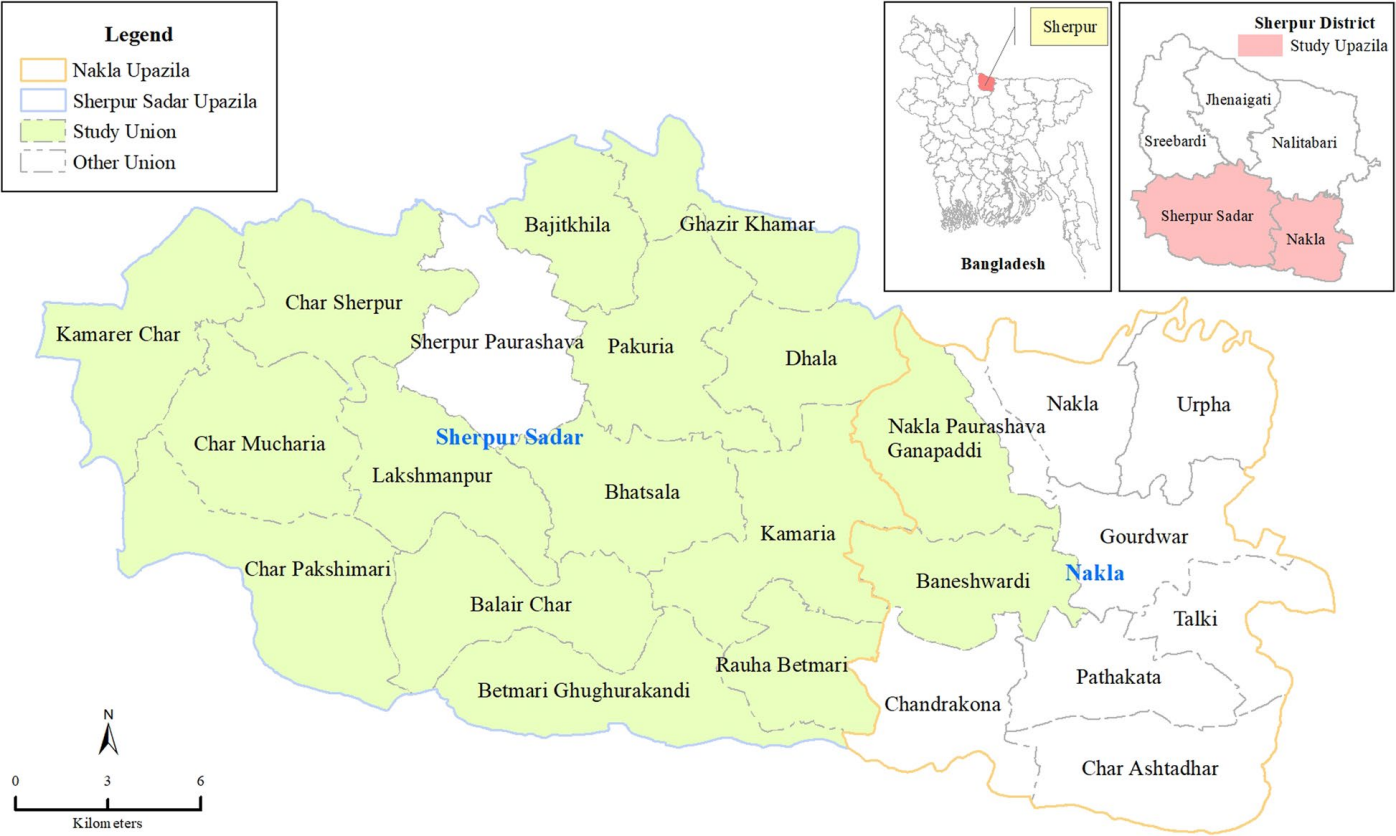
Sherpur, the Poriborton study site, in Northern Bangladesh, divided by Unions (main picture). Insert: Bangladesh divided by Districts, and the selected Sub-Districts (red)

The study will be conducted in the Sherpur District of Mymensingh Division, Bangladesh (Fig. 1, authors own map). The Sherpur district is located approximately 200 km north of Dhaka and has been chosen based on the high neonatal mortality rates and the low saturation of the use of LPG fuel for cooking [25]. Sherpur has a population of ~ 1,170,219 living in rural areas that are divided into five sub-districts. Of these five sub-districts, we have purposively selected two for the study based on the road conditions that allow a good vehicular access with the district headquarters for the transportation of LPG cylinders as well as field team access.

### Eligibility criteria {10}

The Poriborton-Extension Trial has two levels of inclusion and exclusion criteria. Inclusion criteria for this extension part of the trial were mother-child dyads from the Poriborton trial cohort who gave birth to a liveborn infant and who was still alive on or after 15 June 2021 and who gave consent and were willing to continue to participate in the study up to 24 months of the child’s age. Exclusion criteria are women who had a pregnancy loss or stillbirth, the child died before the 15th of June or a livebirth, but the child died before the enrollment visit was made. In case of multiple live births from a pregnancy, all children will be enrolled if they meet the inclusion and exclusion criteria.

### Who will take informed consent? {26a}

Poriborton evaluation staff will recruit mother-child dyads from the original Poriborton trial and obtain informed consent from June 2021.

### Additional consent provisions for collection and use of participant data and biological specimens {26b}

N/A as we do not collect biological specimens for storage.

### Interventions

#### Explanation for the choice of comparators {6b}

The LPG arm will be compared to the control arm (usual cooking practice), to evaluate if reduced exposure to HAP through provision of LPG for cooking from early gestation through to age 2 improves child anthropometry, health, and neuro-cognitive developmental outcomes

#### Intervention description {11a}

The LPG fuel intervention for this extension will commence around birth when the previous cylinder is almost empty. Gas replenishment will continue free of cost until the infant is 24 months old. We have an existing LPG fuel distribution system in operation built into our electronic data collection system. We will continue with the same distribution process with this application. A national gas distribution company will supply the LPG with Bangladesh Standards and Testing Institution certification for cylinder quality. Based on the average duration of use of a cylinder by an average-sized household, we will require approximately 24 refills for each participant until the child is 24 months of age.

**Fig. 2:**
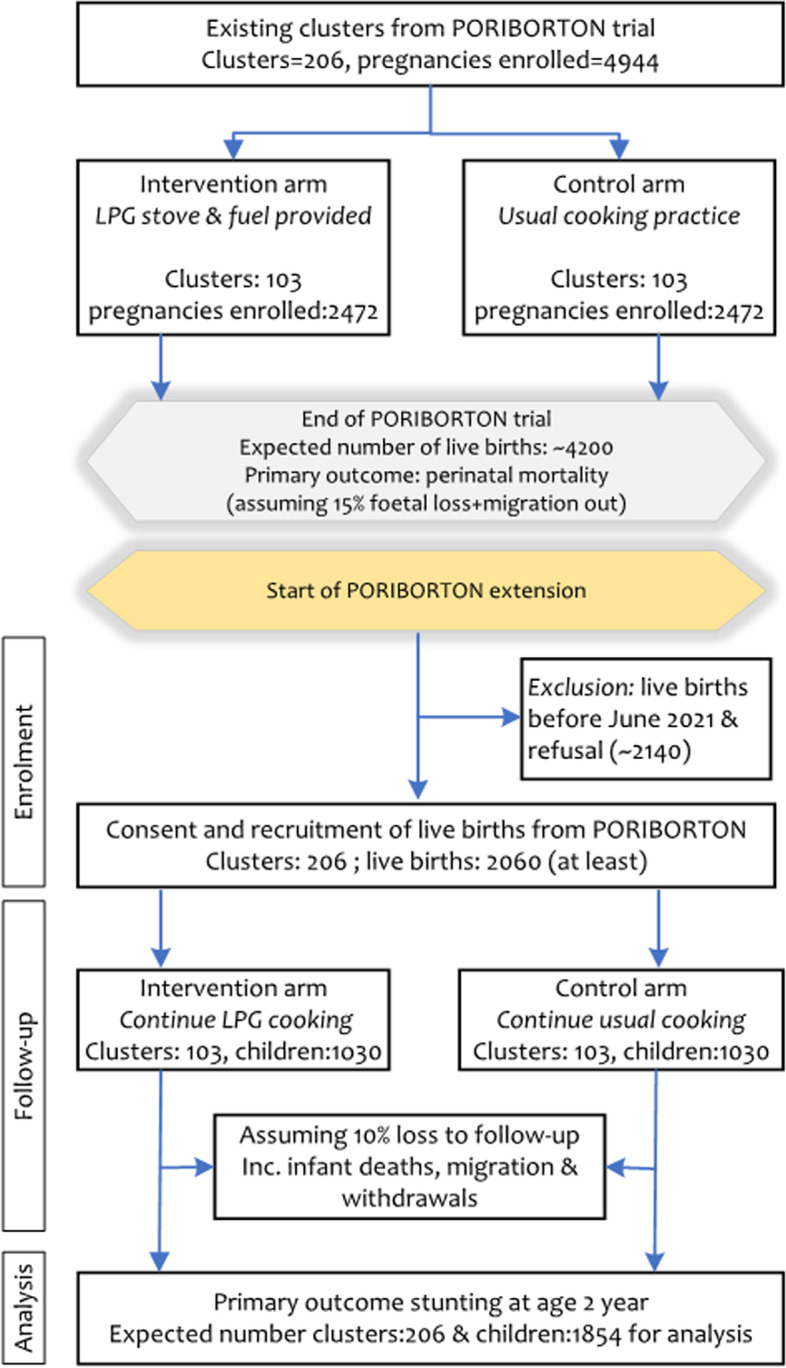
Flow diagram of Poriborton and Poriborton-Extension

The intervention is distributed at the individual level but randomized at the cluster level to reduce community tensions and overall exposure and leverage community-level adoption of clean cooking. We will conduct outcome assessments at the individual level. Allocation concealment is impractical due to the cluster design and nature of the intervention. Participants and their households receive the intervention based on their cluster of residence (Fig. 2).

Our field implementation team is trained in technical issues for gas stove installation, troubleshooting and maintenance, and cylinder replacement. They will continue to provide support to intervention households who require external assistance as necessary. These stoves will adhere to the Bangladesh Standards and Testing Institution requirements. We will replace the stoves in case of mechanical faults during the 2 years of use. Control women will continue with usual cooking practices (mostly traditional cooking) and will be followed up for outcome evaluation up to 24 months of child’s age. Participants from the control arm will be provided 200 Taka as time compensation during the six visits (3, 6, 12, 18, 24 months of child’s age).

#### Criteria for discontinuing or modifying allocated interventions {11b}

The icddr,b’s ethics review committee requires reporting of all serious adverse events within 24 h; for this study, all adverse events will also be reported by the study investigators within 24 h of their occurrences using a standard case reporting form. We anticipate minimal risk to the participants from the study interventions. The quality of gas and stoves are ensured according to the Bangladesh Standards and Testing Institution standards. To avoid rare accident events from LPG stove, extensive counseling and guidance on safety precautions are provided to intervention participants before installing the stove and every 2 months throughout the intervention.

#### Strategies to improve adherence to interventions {11c}

The field implementation team will conduct regular visits (every 2 months) to support intervention households and answer any queries as needed. Based on extensive formative research, we have developed behavior change communication materials that encourage, promote, and support the safe, proper, and consistent use of LPG for cooking. The materials include calendars, leaflets, and short videos.

#### Relevant concomitant care permitted or prohibited during the trial {11d}

N/A as there are no relevant interventions prohibited.

#### Provisions for post-trial care {30}

If the results are favorable to the intervention, we will work with relevant stakeholders to ensure access to the intervention for all communities; these discussions have already commenced.

#### Outcomes {12}

The primary outcome for this trial will be the proportion of stunted children (height-for-age < − 2 Z) at 24 months in the intervention arm compared to the control arm.

Secondary outcomes include the following: (i) mean height-for-age *Z* scores, (ii) mean weight-for-length *Z* scores and prevalence of wasting (weight-for-length < − 2 Z) in children, (iii) mean absolute height-for-age differences, health outcomes: (iv) number of events and mean days with diarrhea, acute respiratory illness and fever, at 6, 12, 18, and 24 months of child’s age, (v) differences in Bayley Scales scores in cognitive, motor and language domains, and Wolke’s behavior ratings during Bayley test (vi) differences in household income/expenditure, (vii) cost of delivering the intervention and costs of upscaling, and (viii) cost-effectiveness of the intervention compared to usual cooking providing estimates of the incremental cost per stunted child prevented and incremental cost per disability-adjusted life years averted, (ix). Intermediate outcomes include PM2.5, 24-h averaged mean, and any differences in gender roles (self-efficacy, time use).

#### Participant timeline {13}

In the first year of this five-year study, we will recruit mother-child dyads from Poriborton close to the birth to ensure the continuation of LPG supply in the intervention arm. We commenced recruitment in 2021, and it will take up to 3 years for the whole cohort of children to reach 24 months of age to complete follow-up. Data cleaning and preliminary analysis will be substantial and will require a year. Field activities will wind down in year 4. Final analyses and manuscript preparation will be moved to Sydney in year 5.

#### Sample size {14}

We expect the final sample size to be at least 1854 children (927 in each arm) with follow-up up to 24 months of child’s age available for analysis. This assumes ten per cent loss-to-follow-up due to mortality in the first 2 years of age (3%), migration out (3%), and withdrawals (2%). For this sample size, we have at least 88% power to detect a 20% lower risk (relative risk =0.8) in stunting, assuming 35% stunting in the control vs 28% in the intervention, an intraclass cluster correlation of 0.02, which is based on the previous cRCT on stunting, [26] and a two-sided significance level of 0.05. If the intraclass correlation is twice as large, i.e., intraclass correlation 0.04, [25], we would still have 80% power. For the secondary outcomes, the final sample size will provide at least 80% power to detect moderate effect sizes for many, if not all, secondary outcomes. For example, we will have 90% power to detect a standardized difference of 0.2 between arms for height-for-age *z*-score. Any differences in perinatal outcomes from Poriborton will not impact the integrity of the extension. We expect reduced perinatal mortality and higher birth weights. Additional live births will result in a slightly higher sample size in the intervention arm for the extension. We investigated this scenario in our power calculation and found the differences in cluster size will remain relatively small and less likely to impact the power.

For the personal exposure of household air pollution, a sample size of 120 children from each arm will be used to estimate reductions in PM2.5. This sample size will allow us to detect a 28% difference (50% and 70% in intervention and control, respectively) (WHO target [3]) with > 80% power.

The expected number of live births (> 7 days old) eligible is 2154, corresponding to all women enrolled in Poriborton from October 2020, of which we expect 10% fetal loss and up to 5% lost-to-follow-up due to migration (3%) or withdrawal (2%). Of the 2154 births (infants), we expect 2060 to be enrolled into the Poriborton-Extension, conservatively assuming that 4% of the newborns die prior to enrollment or decline to participate in the extension.

#### Recruitment {15}

The Poriborton Extension Trial will enroll mother-child dyads from the original Poriborton trial. This extension phase will require a sample size of 1854 which is a subset of the original trial. We will enroll women who give birth to a liveborn infant who survives to or from 15 June 2021, and this will give us the required sample size for this extension trial.

### Assignment of interventions: allocation

#### Sequence generation {16a}

We will use the existing clusters and randomization allocation from the Poriborton trial. These clusters were computer generated using random sequences. In Poriborton, we selected 206 clusters from 16 selected unions of 2 sub districts. The clusters were then randomized with a ratio of 1:1 into LPG and control arm, with 103 clusters in each arm.

The ratio of clusters per arm in each block will be 1:1 for intervention vs. control arm. For Poriborton, we generated random blocks of sequential 1 and 2 using Stata SE, where each number represented either the intervention or control arm. Non-investigators then allocated the clusters to the treatment arms based on the random sequence.

For the Poriborton Extension Trial, we will continue with the same mother-child dyads from the original randomization of the Poriborton trial for this extension of the trial (Fig. 2).

#### Concealment mechanism {16b}

This is a cluster design and concealment, and blinding is impracticable. Field evaluation staff will be blinded to the hypothesis, and we will conduct our analysis blinded to the treatment arms.

#### Implementation {16c}

An independent statistician will generate the allocation sequence. All participants in the intervention clusters will continue receiving the intervention from the original Poriborton trial, and all participants in the control arm continue their usual cooking practice and do not receive any intervention. To minimize bias, since participant blinding is not possible, we have separate intervention and evaluation staff. Trial evaluators will be blinded to the hypothesis, and we will conduct our analysis blinded to the treatment arms.

### Assignment of interventions: blinding

#### Who will be blinded {17a}

This is a cluster design and concealment, and blinding is impracticable. Field evaluation staff will be blinded to the hypothesis, and we will conduct our analysis blinded to the treatment arms. Separate field staff teams are in operation for the intervention implementation and assessment measurement, which prevents measurement bias.

#### Procedure for unblinding if needed {17b}

The design is open label with only outcome assessors being blinded so unblinding will not occur. Blinding is not possible for the intervention and outcome assessment teams as we are giving a stove and LPG cylinders to the intervention participants, however the outcome assessors and data collectors will not be informed of the outcome for the Poriborton extension phase.

### Data collection and management

#### Plans for assessment and collection of outcomes {18a}

Separate field staff teams are in operation for the intervention implementation and assessment measurement, which prevents measurement bias.

##### Child anthropometry

Trained research assistants will collect anthropometric data (weight and height measurements) using in-field electronic data capture on tablets and established methods [27], which will be standardized before and during data collection. Anthropometry will be collected at 6, 12, 18, and 24 months of child’s age. We will use the WHO Growth Standard [28] to construct anthropometric indices and standard indicators, including stunting (height-for-age < − 2 Z), wasting (weight-for-height < − 2 Z), and underweight (weight-for-age < − 2 Z). Children with very low weight-for-height (Z < − 3) will be referred for assessment and treatment for severe acute malnutrition and retained in the study.

##### Bayley Scales of Infant and Toddler Development (IV) and Wolke’s behavior rating

Appropriately qualified and trained staff will conduct the Bayley tests as per the manual. The three key developmental domains include cognition, language, and motor will be assessed with the Bayley Scale. In addition, we will directly rate children’s five behaviors (approach, activity, emotional tone, cooperativeness, and vocalization) using the 9-point scale of the Wolke’s behavior rating during the Bayley test. Our team has expertise in using these tools in community trials in Bangladesh, and until now, several versions of Bayley have been translated, adapted, and used in Bangladesh [29, 30]. This time we will use Bayley - IV, which is the most updated edition and more culturally appropriate. We will collect data at age 12 and 24 months.

##### Infant and young child feeding practices

Infant and young child feeding (IYCF) practices are an important determinant of child growth and development. We will collect IYCF information by a 24-h recall questionnaire used in the national survey and several previous longitudinal studies trials [31, 32]. IYCF data will be collected at 3, 6, 12, 18, and 24 months of child’s age.

##### Family care indicator

To evaluate the amount of stimulation the child receives at home, we will use the family care indicator developed by UNICEF and initially validated in Bangladesh by our team. These questions will be asked to mothers/caregivers at the time of the Bayley test and will pick up information about child and caregiver interactions as well as availability of stimulation/play materials at home [33].

##### Food security

Food security will be assessed at 3, 12, and 24 months using the locally adapted household food security assessment tool [34]. We used a locally adapted standard Household Food Insecurity Access Scale tool [35]. We are currently implementing this into our data collection for the original trial.

##### Respiratory symptoms

The difference in respiratory symptoms in children and their elder siblings (spill-over effect, in children aged > 2) between the two arms is defined as the number of annual cough episodes lasting ≥ 4 weeks [36] and collected at 6, 12, 18, and 24 months using a validated questionnaire [37].

##### HAP exposure

We will measure personal HAP exposure in two ways: using our (1) structured questionnaires used in Poriborton [38], which include smoking in the home, and other known household air pollution variables, and (2) particulate matter: continuous by nephelometry and time-averaged filter-based measurements of particulate matter PM2.5 concentrations. Also, time-averaged black carbon, brown carbon, and tobacco smoke mass concentrations from the filter [39]. The Enhanced Children’s MicroPEM (ECM) is designed specifically for children aged less than 5 years. For children who are not mobile and are carried by their mother, the ECM will be carried by mothers. Once the children are mobile, the device will be worn by the child. Thornburg and his team have used this successfully and safely in South Africa and Malawi. It weighs ~ 150 g and is worn in a culturally appropriate pouch (that we developed) and collected at 6, 12–16 months, and 24 months of age.

##### Gender roles

The impact on gender roles is an essential consideration in understanding the impact of Poriborton-Extension. Women are usually responsible for household cooking, and women are disproportionately exposed to fumes from biomass stoves due to these responsibilities [40]. We will evaluate the impact of the intervention to changes in gender roles, i.e., shifts in the division in of labor, self-efficacy, woman’s access to and control over resources, altered work and time usage (due to less time collecting biomass fuels), household decision-making or bargaining power, and domestic violence. We will use specifically tailored tools, including relevant components from the Project-Level Women’s Empowerment in Agriculture Index [41] and an adapted version of the Clean Cooking Alliance’s “Follow-up In-person User Social Impact Survey” [42]. We will collect data at the 6 months and 18 months points of the intervention.

The timeline for scheduled visits and assessments for the Poriborton Extension Trial is found in Table 1.

**Table 1:** Schedule visit and assessments of the Poriborton Extension Trial

	Tool	Birth	7–10 day	3 months	6 months	12 months	18 months	24 months
**Anthropometry**								
Length	Anthro	Y	Y	Y	Y	Y	Y	Y
Weight	Anthro	Y	Y	Y	Y	Y	Y	Y
MUAC	Anthro					Y	Y	Y
Head circumference	Anthro	Y	Y	Y	Y	Y	Y	Y
**Development**								
Bayley scores	Bayley’s Scales (IV) of Infant &Toddler Development and Wolke’s behavior rating					Y		Y
Family care indicator	UNICEF’s Family Care Indicator tool					Y		Y
**Child feeding**								
Initiation of Breastfeeding	Post-birth	Y	Y (if not birth)	Y (if not earlier)				
Prelacteal feeding	Child feeding tool		Y	Y (if not earlier)	Y	Y	Y	Y
Breastfeeding	Child feeding tool		Y	Y	Y	Y	Y	Y
Feeding other foods and drink	IYCF feeding tool		Y	Y	Y	Y	Y	Y
Feeding frequency	IYCF feeding tool		Y	Y	Y	Y	Y	Y
**Maternal dietary intake**								
Dietary diversity only	Dietary take tool			Y			Y	
**Household food security** (structured tool)	Food security assessment tool			Y		Y		Y
**Morbidity**								
Child morbidity (2-week recall)								
COPD for mothers	COPD assessment tool (CAT) for adult				Y	Y	Y	Y
Respiratory symptoms Children	ARI assessment				Y	Y	Y	Y
**HAP**								
HAP exposure (children)	Existing tool				Y	Y (12–16)		Y
**Gender**								
Gender questionnaire (women)	Adapted Pro-WEAI and CCA User Social Impact Survey				Y		Y	

##### Process evaluation

We will collect mixed-methods data to assess protocol adherence, LPG continued and exclusive and correct use, community engagement, awareness and knowledge of benefits and correct usage of LPG, and contextual factors for adherence and non-adherence of exclusive and continuous use of LPG. Data will be collected at regular time points using quantitative (regular project monitoring records/data) and qualitative methods (interviews and focus groups of a sub-sample of women and household members, and through direct observation). This data will assist with understanding adherence to the intervention (intensity of the intervention) and barriers and facilitators to clean cooking adoption, which will assist with identifying contextual factors influencing the trial outcomes, interpreting our trial findings, and also provide insight for replicating and scale-up.

### Plans to promote participant retention and complete follow-up {18b}

Our intervention is a high-value intervention and is itself a method to promote participant retention and complete follow-up. As this is an extension of the original trial we already know that participants are willing to continue with the intervention. Participants in the control arm receive small gifts (child blankets, toys and transport costs reimbursed) to promote retention and complete follow-up. Data will not be collected from those who discontinue with the intervention.

### Data management {19}

All electronic data is stored on a password secured server backed-up at icddr,b, that only limited staff can access. Data will be stored for 15 years as per trial sponsor funding rules. We will adhere to good data management practices, including collection, validation, and training. Our data will be publicly available as per international standards after 15 years of the trial completion.

### Confidentiality {27}

We have gate-keeper consent from community leaders and relevant government officials. All women and or their guardians provide informed consent to participate and have the right to withdraw at any stage without penalty or loss. The investigators will ensure the privacy, anonymity, and confidentiality of the information provided by respondents and will store all trial information in an encrypted database with all identifiers removed.

### Plans for collection, laboratory evaluation, and storage of biological specimens for genetic or molecular analysis in this trial/future use {33}

N/A as this trial does not collect biological specimens.

### Statistical methods

#### Statistical methods for primary and secondary outcomes {20a}

The main analyses will be by intention to treat. Analyses will be conducted at the infant level but will be adjusted for the cluster randomization using a random effect for clusters in all model development [43]. We will use linear models for continuous outcomes (e.g., height-for-age *Z*-scores) and logistic models for binary outcomes. Bayley developmental scores will be used mostly as continuous variables after converting into both composite scores from conversion tables and *Z*-scores using the mean and standard deviation of control group children across age bands. Functional capacity index and behavior scores will be summed up and compared between arms. Models will include treatment group as a fixed effect and a random effect for the cluster randomization. For data where we have repeated measurements will also include time as a fixed effect and infants as a random effect to account for repeated measurements (three-level mixed models). The balance of household characteristics will be assessed between arms, and secondary analyses will be conducted, adjusting for any imbalanced characteristics. We will use Stata for all analyses.

#### Interim analyses {21b}

N/A as based on previous trial we anticipate minimal risk to participants.

#### Methods for additional analyses (e.g., subgroup analyses) {20b}

In the PM2.5 sub-sample, we will analyze the association of household air pollution (measured by PM2.5) by replacing treatment group with log (PM2.5): PM2.5 will be examined as a continuous exposure variable, but a secondary analysis will also examine the effect of exposure where the PM2.5 exposure variable is categorized by the WHO critical value. We will use Stata for all analyses.

#### Methods in analysis to handle protocol non-adherence and any statistical methods to handle missing data {20c}

We will do a complete case analysis and to avoid biasing the results or excluding data we will apply missing value imputation and undertake sensitivity analysis.

#### Plans to give access to the full protocol, participant level-data and statistical code {31c}

All data will be accessible to the study investigators and will have the right to analyze and publish data. The datasets generated and/or analyzed during the current study are not publicly available at present; however, all data will be available for consideration for sharing from 2025. Data will be available from the corresponding author on reasonable request, with the applicant needing to provide a methodologically sound proposal and subject to approval by the principal investigator with requirements to sign a data access agreement. These procedures are in alignment with the Australia New Zealand Clinical Trials Registry Data Sharing Statement.

### Oversight and monitoring

#### Composition of the coordinating center and trial steering committee {5d}

There are no other committees responsible for this trial. Investigators and field management staff meet weekly.

#### Composition of the data monitoring committee, its role and reporting structure {21a}

For this study all serious adverse events need to be reported by study investigators to icddr,b’s ethics review committee within 24 h of their occurrences using a standard case reporting form. These will be monitored by the relevant committees will monitor any events and based on advice we will not establish a data monitoring committee.

#### Adverse event reporting and harms {22}

The icddr,b’s ethics review committee requires reporting of all serious adverse events within 24 h; for this study, all adverse events will also be reported by the study investigators within 24 h of their occurrences using a standard case reporting form. Poriborton Extension is a low-risk intervention, and as part of routine monitoring, we will conduct refresher training on all safety procedures relating to the use of the stove. Potential minor adverse events may include burning while cooking, but we do not expect this will be an elevated risk compared with normal cooking practices

#### Frequency and plans for auditing trial conduct {23}

The project management group (principal investigators and field team) will meet weekly via zoom and review the conduct of trial in detail as needed. This team will manage trial organization, oversee participant safety, study design, database integrity, and study conduct. There is no procedure for additional auditing.

#### Plans for communicating important protocol amendments to relevant parties (e.g., trial participants, ethical committees) {25}

Any changes to the protocol will be communicated to the ethical review committees at icddr,b and The University of Sydney, as well as the Australian New Zealand Clinical Trials Registry (ACTRN12618001214224).

#### Dissemination plans {31a}

Findings from this study will be shared with stakeholders in Bangladesh with dissemination sessions in-country and with (virtual) global audiences. We will also present the findings at international conferences and publish in conference papers and international peer-reviewed journals. We will distribute our findings using appropriate methods, such as community meetings. If the results are favorable to the intervention, we will work with relevant stakeholders to ensure access to the intervention for all communities, these discussions have already commenced.

## Discussion

Evidence of the effect of air pollution exposure beyond acute disease in children is accumulating, and reduced growth during childhood and developmental impacts are suspected [8, 12], although the specific effect of household air pollution on these outcomes has not been investigated. This trial will make a major contribution to the evidence of the effect of household air pollution exposure on these child health outcomes at age 2. As this is an extension of an existing trial that originally recruited women in early pregnancy, we will be able to assess the effect of reduced exposure during fetal development compared to those children who were exposed during development, through to age 2.

The strengths of this trial include the high-quality cluster randomized controlled design, the existing trial infrastructure including the field processes and procedures that are already established and tested, and the long-term follow-up of the study. Importantly we have developed excellent community relationships, and the community is very supportive of the trial and the continuation of the trial intervention. Before we applied for funding in late 2019, the community was suggesting to our field staff that we should continue the intervention as they thought that it would be even more important to assess the impact on the health on the infant. We are very pleased to conduct a study for whom the intended beneficiaries are supportive.

## Trial status

PR-17103/Version 3/9 July 2021: A cluster randomized controlled trial of cleaner cookstoves to reduce adverse pregnancy outcomes and growth and development at 2 years of child’s age in rural population of Bangladesh. We began recruitment to Poriborton-Extension in August 2021, almost immediately following our award notification. The submission of our protocol was as fast as we could considering the very tight timeline between grant award, study population eligibility and finalizing the protocol. We anticipate recruitment to be completed by approximately end of January 2022, and it was completed in February 2022—after submission of this protocol.

## Data Availability

All data will be accessible to the study investigators and will have the right to analyze and publish data. The datasets generated and/or analyzed during the current study are not publicly available at present; however, all data will be available for consideration for sharing from 2025. Data will be available from the corresponding author on reasonable request, with the applicant needing to provide a methodologically sound proposal and subject to approval by the principal investigator with requirements to sign a data access agreement. These procedures are in alignment with the Australia New Zealand Clinical Trials Registry Data Sharing Statement.

## Abbreviations

HAP: Household air pollution
LMIC: Low and middle-income countries
LPG: Liquid petroleum gas
ECM: Enhanced Children’s MicroPEM
PM: Particulate matter
WHO: World Health Organization
IYCF: Infant and young child feeding
